# A MIQE-Compliant Real-Time PCR Assay for *Aspergillus* Detection

**DOI:** 10.1371/journal.pone.0040022

**Published:** 2012-07-10

**Authors:** Gemma L. Johnson, David F. Bibby, Stephenie Wong, Samir G. Agrawal, Stephen A. Bustin

**Affiliations:** 1 Blizard Institute of Cell and Molecular Science, Queen Mary University, London, United Kingdom; 2 Department of Haemato-Oncology, St Bartholomew’s Hospital, London, United Kingdom; 3 Division of Infection, Barts and the London NHS Trust, London, United Kingdom; 4 Queen Elizabeth Hospital, Kowloon, Hong Kong, Special Administrative Region, People’s Republic of China; University of Aberdeen, United Kingdom

## Abstract

The polymerase chain reaction (PCR) is widely used as a diagnostic tool in clinical laboratories and is particularly effective for detecting and identifying infectious agents for which routine culture and microscopy methods are inadequate. Invasive fungal disease (IFD) is a major cause of morbidity and mortality in immunosuppressed patients, and optimal diagnostic criteria are contentious. Although PCR-based methods have long been used for the diagnosis of invasive aspergillosis (IA), variable performance in clinical practice has limited their value. This shortcoming is a consequence of differing sample selection, collection and preparation protocols coupled with a lack of standardisation of the PCR itself. Furthermore, it has become clear that the performance of PCR-based assays in general is compromised by the inadequacy of experimental controls, insufficient optimisation of assay performance as well as lack of transparency in reporting experimental details. The recently published “Minimum Information for the publication of real-time Quantitative PCR Experiments” (MIQE) guidelines provide a blueprint for good PCR assay design and unambiguous reporting of experimental detail and results. We report the first real-time quantitative PCR (qPCR) assay targeting *Aspergillus* species that has been designed, optimised and validated in strict compliance with the MIQE guidelines. The hydrolysis probe-based assay, designed to target the 18S rRNA DNA sequence of *Aspergillus* species, has an efficiency of 100% (range 95–107%), a dynamic range of at least six orders of magnitude and limits of quantification and detection of 6 and 0.6 *Aspergillus fumigatus* genomes, respectively. It does not amplify *Candida*, *Scedosporium*, *Fusarium* or *Rhizopus* species and its clinical sensitivity is demonstrated in histological material from proven IA cases, as well as concordant PCR and galactomannan data in matched broncho-alveolar lavage and blood samples. The robustness, specificity and sensitivity of this assay make it an ideal molecular diagnostic tool for clinical use.

## Introduction

Real-time PCR (qPCR) is firmly established as the method of choice for the detection of pathogen-derived nucleic acids in routine clinical diagnostics [1]. Several features make qPCR well suited to the clinical environment:

- Speed: assay reaction times are typically measured in tens of minutes
- Convenience: the homogenous assay format obviates the need for post-amplification processing
- Simplicity: the assay requires two primers, an optional probe and a single enzyme
- Sensitivity: single copy targets can be detected, if not quantified
- Specificity: a well-designed assay is specific for a single target, but mismatch-tolerant assays are easily designed
- Robustness: a well-designed assay will yield results across a wide range of reaction conditions
- High throughput: thousands of reactions can be carried out on a single run
- Quantification: the dynamic range is typically huge (eight to nine orders of magnitude)
- Familiarity: PCR has been around for many years and its advantages and disadvantages are well understood
- Cost: assay reagents are inexpensive; together with the trend towards smaller reaction volumes the costs per assay are low.

There are three main drawbacks to qPCR assays: (i) their sensitivity to environmental inhibitors that are concentrated along with pathogens during sample processing can lead to false-negative results – this is important in fungal PCR where large volumes of starting material are often tested, increasing the opportunity for concentration of inhibitors, as highlighted by the European Aspergillus PCR Initiative (EAPCRI) recommendations to extract from at least 3 ml of whole blood and elute DNA into less than 100 µl [2], (ii) assays determine only total pathogen number and do not provide information about whether a pathogen has the ability to establish an infection or not and (iii) variable assay conditions and ill-defined assay designs can generate significant inter-laboratory variation, leading to unreliable and often contradictory results obtained from the same samples.

PCR has been extensively used as an aid in the diagnosis of IA [3], one of the leading causes of mortality in patients with haematological malignancies receiving intensive chemotherapy and haematopoietic stem cell transplantation [4], [5]. *Aspergillus fumigatus* accounts for approximately 90% of all cases of life-threatening IA [6]. However, there has been an increase in the frequency of other *Aspergillus* species associated with IA, with reported frequencies of *A. flavus, A. terreus, A.nidulans* and *A.niger* of 14–33%, 3–28%, 1–2.5% and 5–8%, respectively [7]–[9]. Therefore, a reliable diagnostic assay for IA should detect all *Aspergillus* species whilst not amplifying other, clinically significant non-*Aspergillus* targets, such as *Candida* spp.

A lack of technical standardisation and poor understanding of the kinetics of *in vivo* fungal DNA release has resulted in the development of numerous assays amplifying diverse target regions and reporting a wide range of PCR sensitivities and specificities [2]. A recent systematic review of *Aspergillus* PCR methods using whole blood samples [10] highlighted the variation in methods across 16 studies that included three extraction and four disruption methods, three different starting volumes, three different specimen types, three different target genes and four different PCR methods. Only four qPCR assays were included in this review and there was sufficient information to determine PCR efficiency for only two – both suboptimal at 91.6% and 86%. This, together with the inadequate reliability of amplification from blood and serum has led to attempts to reach a consensus on PCR diagnosis. This was pioneered by EAPCRI, which aims to standardise *Aspergillus* PCR [2]. The authors circulated quality control panels to 24 centres for evaluation of their existing methods. A simple inspection of the data reveals the widely differing sensitivities of the different assays, with detection thresholds ranging over three orders of magnitude (0.27 to 270 copies/µl). There was also variation between laboratories carrying out the same assay, with results differing by up to 10-fold. To address all these issues, we have applied the MIQE guidelines for qPCR assay design and reporting [11]–[13], which have been a defining event in the maturing of qPCR technology, and report the design of a state-of-the-art qPCR assay for diagnosis of IA. Clinical evaluation of the assay was performed using histological samples from proven IA cases and broncho-alveolar lavage (BAL) fluid and blood from adults at high risk of IFD following intensive chemotherapy or allogeneic stem cell transplantation.

## Results

### Assay Characterisation

#### Primers and amplicon

The primer annealing sites are free of significant secondary structure and primers amplify a 76 base pair (bp) amplicon with no similarity to the human genome. There is no significant similarity with other clinically relevant fungal pathogens except for *Penicillium* species.

#### Annealing temperature

Temperature gradient analysis of the primers in a reaction with *A. fumigatus* DNA template established a wide optimum primer annealing temperature range of 55–61.4°C (Table 1). The annealing temperature was set at 59°C for subsequent reactions.

**Table 1:** Temperature gradient analysis.

Annealing temperature (°C)	C_q_ values (cycles)
65	29.5
64.5	27.2
63.3	25.1
61.4	24.2
59	24.1
57	24.2
55.7	24.5
55	24.2

Temperature gradient analysis of the primers with *A. fumigatus* DNA template. Analysis performed using SYBR Green.

#### Analytical Specificity

**Figure 1:**
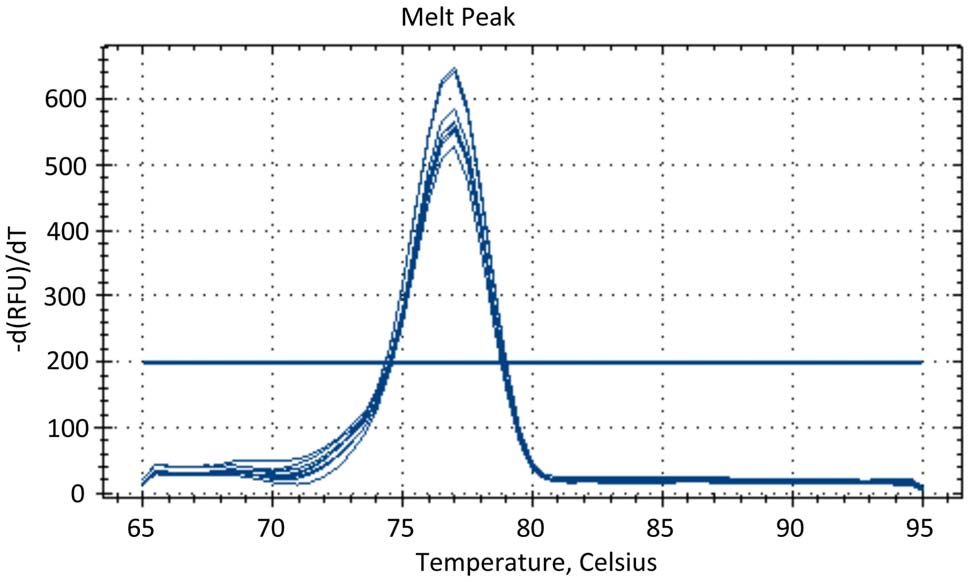
Dissociation (melt) curve analysis of amplification products using *A. fumigatus* genomic DNA dilutions of 2 ng (6×10^4^ genomes), 200 pg (6×10^3^ genomes) and 20 pg (6×10^2^ genomes). Melting temperature of 77°C.

*A. fumigatus* DNA was extracted from a clinical isolate, which was authenticated by MALDI-TOF analysis. All other DNA samples were prepared from typed cultures. The assay detects *A. flavus. A. niger, A. terreus* and *A. nidulans* DNA; it does not detect *C. albicans, C. dubliniensis*, *S. prolificans, F. solani* and *R. oryza*e DNA. No amplification was seen with human DNA or in any non template control (NTC) well. Assay specificity was validated by melt curve analysis of *A. fumigatus* genomic DNA (Figure 1), indicating a melting temperature of 77°C for the target amplicon. PCR products were analysed using the Lab901 ScreenTape, confirming the correct amplicon size of 76 bp. PCR products were also sequenced, confirming amplification of target sequence.

### Clinical Evaluation

The qPCR assay was evaluated using histological material from three patients with haematological malignancies, who had received intensive chemotherapy, loaded in duplicate wells (Table 2). A post-mortem lung biopsy (sample A) and an ante-mortem brain biopsy (sample B), from two patients with proven IA, amplified with mean C_q_s of 24.6 and 31.5, respectively. Referring to the standard curve, this equates to 35 and two *A. fumigatus* genome equivalents/ng DNA extract, respectively. A control post-mortem lung biopsy with no evidence of fungal infection (sample C), did not amplify.

**Table 2:** PCR results for wax-embedded tissue samples.

Tissue sample	Diagnosis	Mean C_q_	DNA load of extract (ng/µl)	*A. fumigatus* genome equivalents/ng DNA extract
A	Proven IA	24.6	83	35.5
B	Proven IA	31.5	13	1.8
C	No fungal infection	Negative	172	0

PCR results when testing DNA extracts from histological samples A (post-mortem lung biopsy), B (ante-mortem brain biopsy) and C (post-mortem lung biopsy). Genome equivalent of PCR result calculated from *A. fumigatus* standard curve plot.

The qPCR assay was further evaluated using BAL fluid, loaded in duplicate wells, from 11 adults at high risk of IFD following intensive chemotherapy or allogeneic bone marrow transplant (Table 3). BAL fluid samples from episodes 1–7 and 10 were PCR negative and galactomannan (GM) negative (GM Index <1.0). A total of 50 sera and corresponding EDTA whole blood samples were collected during these episodes. All sera were GM negative. All corresponding whole blood extracts were PCR negative.

**Table 3:** Clinical evaluation of BAL and blood in 11 adults at high risk of IFD following intensive chemotherapy or allogeneic stem cell transplantation.

	EORTC/MSG score		BAL fluid		Blood/serum	
Episode	2002	2008	PCR result (mean C_q_)	GM result	PCR result (mean C_q_)	GM result^i^
1	Possible	Non classifiable	Negative	0.4	Negative	<0.5
2	Possible	Possible	Negative	0.1	Negative	<0.5
3	Possible	Non classifiable	Negative	0.2	Negative	<0.5
4	Possible	Possible	Negative	0.4	Negative	<0.5
5	Possible	Non classifiable	Negative	0.2	Negative	<0.5
6	Possible	Possible	Negative	0.3	Negative	<0.5
7	Possible	Possible	Negative	0.4	Negative	<0.5
8	Probable	Non classifiable	34.8	4.1	NA	NA
9	Possible	Non classifiable	27.8	6.4	A. Negative^iii^,B. Negative, C. 37.6	A. Negative^iii^,B. Negative, C. 2.2
10	Possible	Non classifiable	Negative	0.3	Negative	<0.5
11	Possible	Non classifiable	Positive^ii^	1.41	36.8	0.15

NA  =  no sample available for testing.

i. <0.5 indicates that all samples from serial testing were negative.

ii. C_q_ not stated, as >1 µl was loaded, hence C_q_ is not comparable.

iii. A, B, C represent serial samples (see corresponding text).

BAL fluid samples from episodes 8–9 and 11 were PCR positive and GM positive (GM Index >1.0). Episode 8 had no corresponding sera or EDTA whole blood available for analysis. For episode 9, three EDTA whole blood samples and corresponding serum samples were collected 25 (A), 19 (B) and 8 (C) days prior to BAL fluid sampling and tested PCR negative/GM negative, PCR negative/GM negative and PCR positive/GM positive, respectively.

For episode 11, one EDTA whole blood and corresponding serum sample were collected 19 days prior to BAL fluid sampling and tested PCR positive/GM negative, respectively.

### PCR Efficiency/Analytical Sensitivity

**Figure 2:**
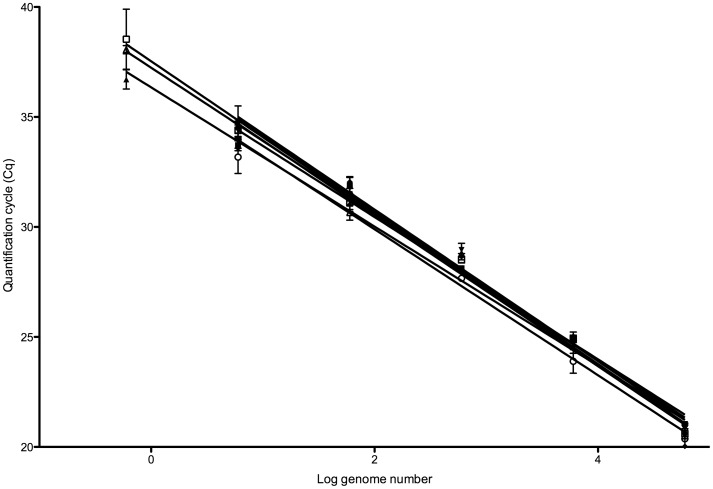
Eight standard curve plots, generated using *A. fumigatus* genomic DNA.

PCR efficiencies were calculated from the slopes of eight standard curves, that were run in duplicate or triplicate on separate plates, incorporating 91 data points (Figure 2). The efficiency range was 95–107%. Dilutions spanned six orders of magnitude from 2 ng (6×10^4^ genomes) to 20 fg (0.6 genomes). 0.6 genomes give rise to an appropriate amplification product in every assay.

### Reproducibility

Reproducibility was excellent across all six orders of magnitude, with an inter-run standard deviation of between 0.37 (for 6×10^4^ genomes load) and 0.97 (for 0.6 genome load). Limits of quantification and detection were calculated as 6 and 0.6 *A. fumigatus* genomes, respectively.

### Inhibition

Each of the DNA extracts from clinical material was included in a SPUD assay and results indicated that no inhibition was present in these samples.

## Discussion

qPCR has become the most widely used molecular technology for diagnostic applications designed to detect and quantify pathogens [1]. *Aspergillus*-specific qPCR assays have been proposed as alternatives to conventional diagnostic procedures for IA, where early diagnosis and treatment are critical [2], [14]–[16]. However, the European Organization for Research and Treatment of Cancer/Mycosis (EORTC) and Mycoses Study Group of National Institute of Allergy and Infectious Diseases (NIAID) do not endorse the routine use of PCR in the diagnosis of IA [17]. There are several reasons for this: (i) disparate chemistries that include the use of SYBR Green [18], although it lacks the required specificity for use in clinical diagnostic assays [19], hydrolysis probes [20]–[22], hybridisation probes [23] and molecular beacons [24], [25]; (ii) variability of targets (28S, 18S and ITS2) [2], [20], [26]; (iii) sample-specific instrument-dependent variability {[10], [27], including contamination-prone nested PCR methods aimed at enhancing analytical sensitivity [23], [28].

*Aspergillus* qPCR assay design has also been hampered by the use of primers designed for conventional PCR assays [23], [26], [29]. This can lead to suboptimal qPCR as the amplicons are relatively large and no structural analyses of primers or primer binding sites have been carried out. These parameters have a strong influence on PCR efficiency and determine analytical sensitivity and reliability of quantification [30]. Hence it is not surprising to note that one report quotes a PCR efficiency of 77% [23], despite amplifying a short region of DNA (91 bp). Our structure analysis [31] of the amplicon revealed a large stem structure at its 3?-end. In a setting that requires low copy number detection, it is vital to have contamination assessment at the DNA extraction stage. Negative extraction controls (no DNA template present) are extracted alongside clinical samples, to detect any cross contamination or environmental contamination. A significant number of studies fail to use or report the use of negative extraction controls to monitor contamination during the extraction stage and, even if they do, they are usually water-only, as opposed to controls that have similar properties to the clinical sample but without target nucleic acid [32]. Ideally, biological (sample) replicates should also be included, especially when the qPCR results suggest very low levels of fungal DNA. Even though it is well known that PCR inhibitors can reduce product yield and even result in complete failure of the PCR, only just over half of the studies evaluated by Khot et al [32] reported the use of some form of an inhibition control, also referred to as an amplification control.

With any assay targeting *Aspergillus*, the risk of procedural contamination is high, and false-positive results may arise due to the ubiquitous nature of fungi in the environment, as well as product carryover from positive samples or from cross-contamination by PCR products from earlier experiments [32]. Therefore, we instituted rigorous procedures for the monitoring of contamination during sample handling, DNA extraction and PCR set up. Reagents were tested for contamination on a regular basis and a separate biosafety cabinet was used for the reaction setup. We included multiple negative controls, i.e. samples that are as similar to the test samples as possible but exclude the target. We also used the SPUD assay [33], as inhibition assessment is mandatory for all clinical diagnostic qPCR assays. The inclusion of such rigorous experimental controls has enabled us to avoid procedural false positivity and false negativity.

We set out to develop an assay that is not just rapid, sensitive, specific and comparable in cost to culture-based diagnostic techniques but that rigorously complies with the MIQE guidelines, both in design and in its reporting. Our assay satisfies all of these criteria essential for a clinically useful molecular assay:

1. Speed: Following extraction of DNA, results from the qPCR assay are available within 60 min. The development of fast PCR reagents, together with the use of smaller reaction volumes and novel thermocyclers has the potential to reduce this to below 5 minutes.
2. Sensitivity: analytical sensitivity is a critical parameter of any diagnostic qPCR assay since the fungal load in the blood of patients with IA is believed to be very low (30–100 fg/µl or less) [21], [23], [34], [35]. Analytical sensitivity refers to the smallest number of nucleic acid molecules that can be detected and distinguished from a zero result. This is best done using a standard curve, which defines the range of the assay and hence the upper and lower target concentration that can be reported. Our assay design targets an amplicon devoid of secondary structure, has been extensively optimised, is linear over at least six logs of template concentration and has an efficiency close to 100%. Its limit of quantification is 6 *Aspergillus fumigatus* genomes, whereas its limit of detection is 0.6 genome copies. Since a single *A. fumigatus* genome contains between 38 and 91 target rDNA sequence copies [36], this suggests our assay can detect as few as 23 target copies [36].
3. Specificity: analytical specificity is determined by identifying the percentage of samples without the target sequence that generate a positive result. If a well-designed assay is used, in the absence of contamination, this will be zero. In a qPCR experiment all detectable products, be they specific or non-specific, contribute to the final amplification plot and hence any qualitative or quantitative result. Post reaction melt analysis using SYBR Green I dye confirmed the specificity of the assay, since it resulted in a single peak and sequencing of the amplicons confirmed the amplification of *Aspergillus* target sequences. Our assay is a pan-*Aspergillus* assay since *Aspergillus fumigatus* is implicated in only up to two thirds of cases of IA, with other *Aspergillus* species, notably *A. flavus* and *A. terreus*, also detected in a significant number of cases [7]–[9]. Importantly, the assay does not amplify human DNA or fungal DNA extracts from *Candida, Fusarium, Scedosporium* or *Rhizopus* species, well-characterised pathogens of immunocompromised (and immunocompetent) patients. Unsurprisingly, since there is 100% sequence identity across the 18S subunits of *Aspergillus* and *Penicillium*, *Penicillium* DNA is also amplified. Infection with *Penicillium* species, specifically *P. marneffei*, is clinically relevant.
4. Cost: Excluding the expense of a thermocycler, the cost of running this *Aspergillus*-specific assay including the use of spin column DNA extraction, qPCR reagent and plastics cost is less than US$ 500 per 96 reactions. In comparison, the Platelia *Aspergillus* galactomannan antigen test (Bio-Rad Laboratories), which is included in the EORTC/MSG criteria for the likelihood of IFD [17] has a list price of approximately US$ 770 for 96 reactions.

We evaluated our qPCR assay on histological material from two cases of proven IA with *Aspergillus fumigatus* (see Table 2). However, in patients at high risk of IFD post chemotherapy or allogeneic stem cell transplantation, it is rarely possible to get diagnostic material to definitively establish IA. Therefore, we evaluated BAL from 11 patients at high risk and found complete correlation between PCR and GM results (see Table 3). Furthermore, although detected at the limits of the assay, *Aspergillus* DNA was only amplified in blood samples of patients with PCR and GM positive BAL fluid. Our data shows discordance with the EORTC/MSG scores, with the three episodes that were PCR and GM positive from BAL fluids scoring as ‘non classifiable’ by 2008 criteria. Whilst the EORTC/MSG criteria are the only accepted tool for the classification of the likelihood of IA in cases where evidence for proven IA is lacking, the limitations of these criteria are documented in the literature [37]. A key change between the EORTC/MSG 2002 and 2008 criteria was the elimination of minor clinical criteria and the emphasis placed on specific computerised tomography (CT) findings. There is increasing evidence to support extending the radiological suspicion of IA to less specific chest CT scan findings when supported by microbiological evidence in high-risk haematological patients [38].

In this report we have developed the first qPCR assay targeting *Aspergillus* species that has been designed, optimised and validated in strict observance of the MIQE guidelines. We have reported all its relevant parameters, which clearly demonstrate the huge potential qPCR has as a tool for the early diagnosis of IA. However, reliable diagnosis of IA depends on at least two factors in addition to the quality of the qPCR. The first is sample quality: sample collection, preparation and transport as well as nucleic acid extraction methods are critical parameters in test performance and must be optimised and, ideally, standardised. In principle, extraction of fungal nucleic acids, especially if present in a cell-free state, from broncho-alveolar lavage fluid, blood and serum is relatively straightforward; however, it is easy to co-purify inhibitors of the PCR that will generate inconsistent and unreliable results. Hence our recommendation for the use of dilution curves for every sample to highlight contamination. Secondly, regular calibration of the real-time instrument is crucial for obtaining consistent and accurate results. C_q_ is neither absolute nor invariant, but varies between assays carried out on different days with different reagents, different users or on different instruments. This is because the C_q_ depends on the instrument’s threshold setting, which in turn depends on background fluorescence, which varies with different probes, chemistries, instruments and assay protocols. Finally it is important to emphasise that a definitive identification of *Aspergillus* in clinical samples is made difficult by the absence of any gold standard [17], [39]. Therefore, an assessment of the clinical utility of this, or any molecular assay, for detection of *Aspergillus*, is challenging [40] and will require prospective studies.

## Materials and Methods

### Ethics Statement

The work was ethically approved by the East London & the City Local Research Ethics Committee 1. Participants were recruited from Barts and the London.

Study title: Early diagnosis of invasive Aspergillosis in a high risk group of patients using serum and bronchoalveolar lavage fluid real time PCR and galactomannan ELISA.

REC reference number: 05/Q0603/68.

Ethics amendment dated 28/10/2009. Approval number ReDA : 003933 QM.

### Assay Characterisation

#### Primers and amplicon

A search of the National Center for Biotechnology Information (NCBI) GenBank sequence database (http://www.ncbi.nlm.nih.gov/genbank/) was performed to obtain accession numbers for the target organisms *A. fumigatus* (Accession no FJ 840490), *A. flavus* (Accession no: D63696), *A. terreus* (Accession no. DQ173743) and *A. niger* (Accession no.: GQ338836). 18S rDNA sequences were imported into the CLC Sequence Viewer (http://www.clcbio.com) and aligned to identify suitable target sequences. Primers and a probe with locked nucleic acid (LNA) modifications were designed by Beacon Designer, version 7.2, selecting for a primer annealing temperature of 55°C and amplicon length of <100 bp (Table 4). *In silico* analysis of amplicon specificity was performed using nucleotide BLAST (www.ncbi.nlm.nih.gov/BLAST/). Target secondary structures and primer/template accessibility were assessed using the MFOLD web server (http://mfold.bioinfo.rpi.edu/) using corrections for ionic conditions of 50 nM Na^+^ and 3 mM Mg^2+^, and a folding temperature of 55°C.

**Table 4:** Oligonucleotide primers and probe used in this assay.

Name	Purpose	Sequence 5′ to 3′
Asp-rv	Reverse primer	5′-CTAACTTTCGTTCCCTGATTAATG-3′
Asp-fw	Forward primer	5′ –CTTGGATTTGCTGAAGACTAAC-3′
Asp-LNA	LNA fluorescent probe	5′FAM- catCctTggCgaAtgct-3′BHQ-1

Upper case letters of probe sequence indicate position of LNA-incorporated oligonucleotides.

#### Empirical PCR optimization

All qPCR assays were performed on a Bio-Rad CFX Real-time system (BioRad, Hercules, CA, USA) in a 96-well plate format (FrameStar®, 4titude Ltd, UK). SYBR Green I chemistry and melt curve analyses were used to determine optimal annealing temperatures using the CFX’s temperature gradient function (50°C–60°C). Optimal primer concentrations (50–400 nM) were determined by identifying conditions resulting in the lowest C_q_ combined with absence of primer dimer formation, with each concentration run in four replicate wells.

Reaction volumes were set at 10 µl. SYBR Green assays contained 1X BioRad iQ SYBR Green Supermix, 200 nM each primer, and 1 µl of DNA extract. Initial thermal cycling conditions were 1 cycle of 95°C for 3 mins, followed by 40 cycles of denaturation at 95°C for 10 sec and annealing/polymerisation with a temperature gradient from 50–60°C for 30 sec. Once optimal conditions were established, hydrolysis probe reactions were run comprising 1X BioRad iQ Supermix, 200 nM of each primer, 100 nM probe, and 1 µl of DNA. Post-run analyses were performed using Bio-Rad CFX Manager version 2.0. Threshold C_q_s were calculated from a baseline subtracted curve fit.

#### Analytical Specificity

Analytical specificity of the qPCR assays was assessed using DNA templates extracted from human tissue as well as from a single isolate of each of *A. fumigatus* (clinical isolate, confirmed by MALDI-TOF*), A. flavus* (NCPF 2008), *A. terreus* (NCPF 2729), *A. niger* (NCPF 2275), *A. nidulans* (NCPF 2181), *Candida albicans* (NCPF 3939), *Candida dubliniensis* (NCPF 3949) and *Fusarium solani* (CBS 224.34), *Scedosporium prolificans* (CBS 100391) and *Rhizopus oryzae* (CBS 112.09). Samples were run in duplicate wells. PCR products were analysed on the Lab901 ScreenTape microfluidics system and by direct sequencing, to confirm amplification of the target sequence.

### Clinical Evaluation

The qPCR assay was further evaluated using histological material from three patients with haematological malignancies and BAL fluid from 11 adults (with contemporaneous EDTA whole blood samples when available) at high risk of IFD following intensive chemotherapy or allogeneic stem cell transplantation. The clinical data and samples of these patients were collected in an ongoing study (see Ethics statement above). After 72 hours of persistent fever unresponsive to antibiotics, a CT scan of the chest was performed and, if abnormal, followed by a bronchoscopy and BAL. Blood and serum sampling was performed twice weekly. Twice weekly serum GM monitoring was performed as part of a screening strategy throughout the episode of intensive chemotherapy.

Using the criteria of the EORTC/MSG [17], [39], two physicians independently scored each episode: 10 patients were classified as possible and one as probable IA using 2002 criteria; whereas with the 2008 criteria, the single probable case and 6 of the 10 possible cases were downgraded to ‘non classifiable’ (see Table 3).

#### DNA extraction of fungal culture

Fungal strains were grown on Sabouraud agar at 37°C. Genomic DNA was extracted according to the in-house molecular microbiology standard operating procedure for extraction from fungal culture (MM-1.3 version 4, 30/9/2007) in the Medical Microbiology Laboratory of the Royal London Hospital. Briefly, 0.3-1 g of 0.5 mm diameter glass beads (BioSpec products) were added to an apex tube with an O-ring screw cap. 180 µL ATL buffer and 20 µl proteinase K (both from QIAamp DNA extraction kit, Qiagen, Germany) were added to the tube, with 2 loopfuls of biomass from the culture plate. The tube was shaken in a mini bead-beater for 30 seconds on the ‘homogenise’ setting (BioSpec products), before being incubated at 56°C for 2 hours, with occasional vortexing. A QIAamp DNA mini kit (Qiagen, Germany) was used to complete the extraction, following the manufacturer’s protocol from the step where AL buffer is added. Elution comprised two 100 µl volumes of AE, with centrifuging between). Extracted DNA was quantified using the Implen Nanophotometer and diluted in Qiagen kit AE buffer to a concentration of 2 ng/µl for PCR testing. A negative extraction control of RNase free water was included in each batch of extractions to monitor contamination.

#### DNA extraction from wax embedded tissue samples

A post-mortem lung biopsy (sample A) and an ante-mortem brain biopsy (sample B), were obtained from two patients who had received chemo-immunotherapy for haematological malignancy, whose biopsies showed proven IA. A control post-mortem lung biopsy with no evidence of fungal infection (sample C), was also obtained.

Shavings were obtained from the three wax embedded histological blocks using disposable blades.

Extractions were carried out in a Class 2 biosafety cabinet, which was cleaned with BioCleanse wipes and DNA Away (VWR, UK) before and after use. Briefly, 1 ml xylene (Sigma Aldrich) was added to a tube containing a 5 mm^2^ section of paraffin wax-embedded tissue. This was incubated with shaking for 5 minutes at room temperature, centrifuged 10,000 *g* for 2 minutes, followed by removing and discarding the supernatant. This entire process was then repeated, with the shaking incubation time increased to 20 minutes. 1 ml absolute ethanol (Sigma Aldrich) was added, and the tube centrifuged at 10,000 *g* for 3 minutes. The supernatant was discarded, and the ethanol step repeated. Samples were air dried for 10 minutes in a laminar flow hood. Tissue pellets were resuspended in 180 µl of ATL buffer and 20 µl proteinase K (both from QIAamp tissue kit, Qiagen, Germany) by pulse vortexing, incubated at 56°C for 2 hours, and boiled for 5 minutes. After cooling to room temperature, the Qiagen QIAamp tissue kit column extraction method was followed according to the manufacturer’s instructions, eluting into 50 µl buffer AE. Extracted DNA was quantified using the Implen Nanophotometer. DNA-free water was run as a negative extraction control in each batch of extractions to monitor contamination.

#### DNA extraction from BAL fluid and EDTA whole blood samples

200 µl of clinical sample was added to a 2ml sample tube with 10 µl proteinase K (both from Qiagen EZ1 DNA tissue kit) and mixed by gentle vortex. This was incubated at 56°C for 15 minutes, then spun down to remove condensation from lid. Extraction was then performed on the EZ1 robot (Qiagen, Germany), using EZ1 DNA tissue card programme and tissue kit, eluting into 50 µl. DNA extracts were stored at −20°C prior to PCR analysis. DNA-free water was run as a negative (fungal free) extraction control in every batch of extractions to monitor for contamination during the extraction process.

### Galactomannan Assay

GM detection was performed by Platelia Aspergillus enzyme immunoassay (PA-EIA, Bio Rad, France). Techniques were carried out as recommended by the manufacturer. Results were expressed as a GM index value – the ratio of the sample optical density (OD) divided by the mean OD of the 2 threshold controls. Serum and BAL fluid samples were scored as positive if the GM index was >0.5 and >1.0, respectively.

### PCR Efficiency/Analytical Sensitivity

2 ng of genomic DNA, corresponding to 6×10^4^ genome copies of *A. fumigatus* (clinical isolate*)* was serially diluted over 6 orders of magnitude in 200 ng of *Aspergillus*-free human DNA per dilution. Using qPCR, standard curves were constructed to calculate PCR efficiency, the linear range of the assay and the limits of detection and quantification. PCR efficiencies were calculated from the slopes of eight standard curves that were run in duplicate or triplicate on separate plates, incorporating 91 data points. Quantification was determined on the basis of one *A. fumigatus* genome containing 30 fg of DNA, calculated from a genome size of 4.29×10^6^ bp [41].

### PCR Setup Controls

Multiple NTCs were included with every assay and amplification of a single NTC well invalidated the entire qPCR run, leading to a repeat run. A positive control containing six genome copies of *A. fumigatus* (clinical isolate) was also included with each run, to monitor inter-assay consistency. Amplification had to be reproducible, occurring in all replicate wells, for a sample to be considered ‘PCR positive’.

### Carryover Contamination Controls

All DNA preparations were carried out in a facility that was physically separate from the qPCR laboratory. A unidirectional workflow pattern (pre- to post-qPCR) was enforced, with physically separate laboratories utilised for pre- and post-qPCR analysis.

Reaction set-up took place in a laminar-flow biosafety cabinet, following thorough cleaning of the cabinet, pipettes and tip boxes with DNA-Away (VWR, UK). Plates were never opened post-PCR.

### Inhibition Testing

The SPUD assay was used as an exogenous amplification control [33]. The SPUD inhibition assay comprises a control qPCR assay in which the SPUD amplicon is the only amplifiable target performed in the presence of water. This generates a reference C_q_ value for the SPUD amplicon, characteristic of an uninhibited assay. If the water is substituted with DNA from a sample, a shift of greater than one cycle, to a higher C_q_ and reduced amplification efficiency indicates the presence of PCR inhibitors in the sample. Each qPCR reaction comprised 1X BioRad iQ Supermix, 400 nM of both SPUD forward and reverse primers, 200 nM SPUD probe, 1 µl of SPUD amplicon, and 1 µl of sample extract. Thermal cycling conditions were 1 cycle of 95°C for 3 mins, followed by 40 cycles of 95°C for 10 sec, 61°C for 15 sec and 70°C for 30 sec.
